# Supplementary material for: Associative learning in larval and adult Drosophila is impaired by the dopamine-synthesis inhibitor 3-Iodo-L-tyrosine
Source: Biol Open. 2021 Jun 9;10(6):bio058198. doi: 10.1242/bio.058198 (PMC8214425; doi:10.1242/bio.058198)
Supplement: Supplementary information [file biolopen-10-058198-s1.pdf]

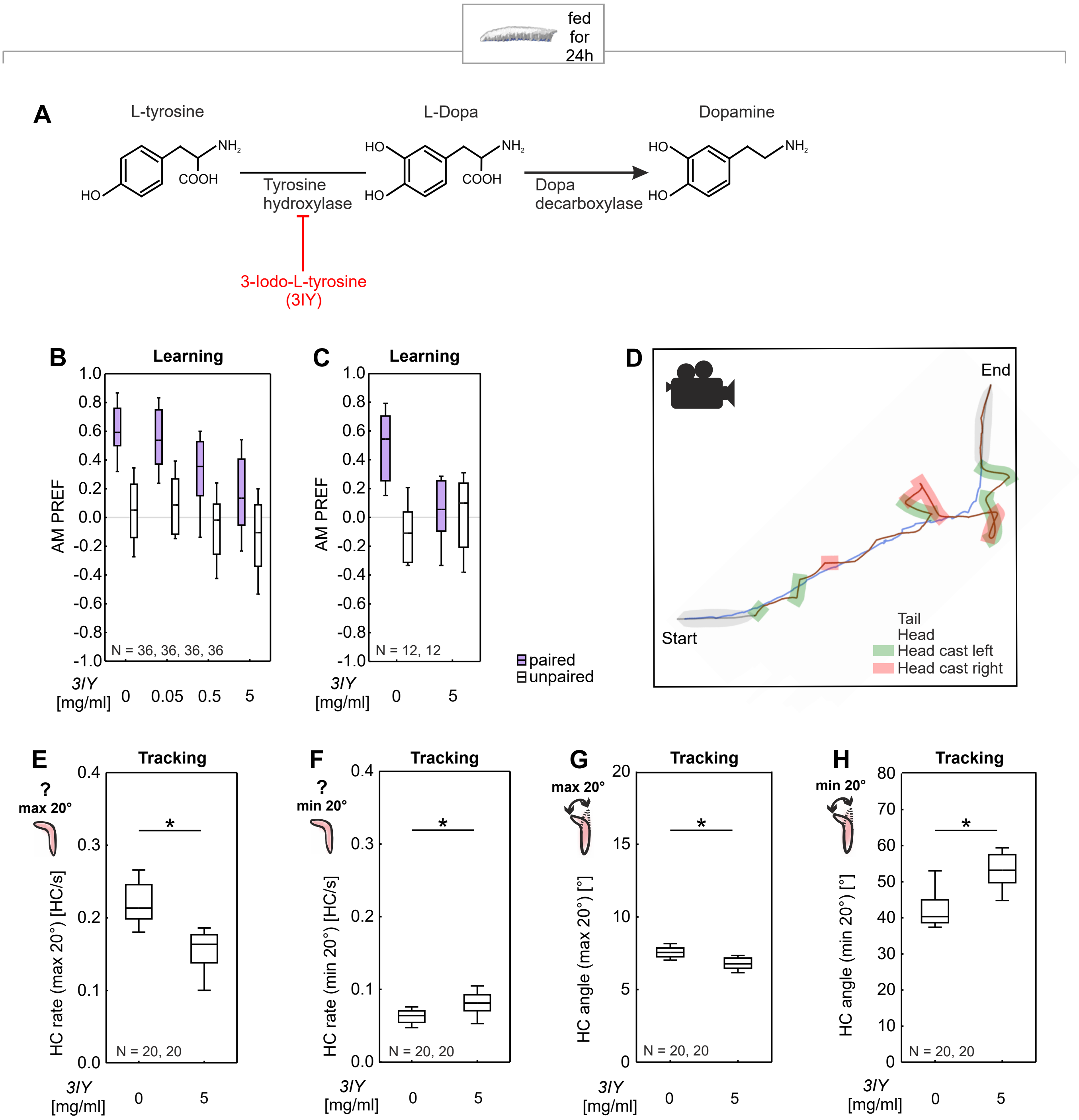

Fig. S1. Preference and tracking data underlying the results shown in Fig. 1. (A) Dopamine is synthesized via two enzymatic steps. In the first step the amino acid L-tyrosine is converted into L-3,4-dihydroxyphenylalanine (L-DOPA) via tyrosine hydroxylase (TH). In the second step, L-DOPA is converted to dopamine via dopa decarboxylase (DDC). In red, the inhibition of the TH enzyme by 3-Iodo-L-tyrosine (3IY) is shown. (B,C) Preference data refer to the Performance Indices shown in Fig. 1B,C, respectively. Purple boxes represent odor preference after paired training, white boxes after unpaired training. (D) Example of a track from the video-recording of a single larva showing relatively straight runs interrupted by lateral head movements (head cast, HC). (E,F) HC rate for small and large HCs, respectively, classified by a HC angle smaller or greater than 20°. This classification as well as the calculation of the HC angle is based on Paisios et al. (2017). The HC rate was decreased for small HCs (E) (MWU: U = 29.00, P < 0.0001, N = 20 each) and increased for large HCs (F) (MWU: U = 85.00, P = 0.0019, N = 20 each) for larvae fed with 3IY for 24 h. (G,H) HC angles classified by small and large HCs. The average HC angle was decreased for small HCs (G) (MWU: U = 47.00, P < 0.0001, N = 20 each) and increased for large HCs (H) (MWU: U = 46.00, P < 0.0001, N = 20 each) for larvae fed with 3IY for 24h. Asterisks above horizontal lines reflect significance in MWU-tests. Box plots represent the median as the midline, 25 and 75% as the box boundaries, and 10 and 90% as the whiskers. Sample sizes are indicated within the figure.

Biology Open • Supplementary information

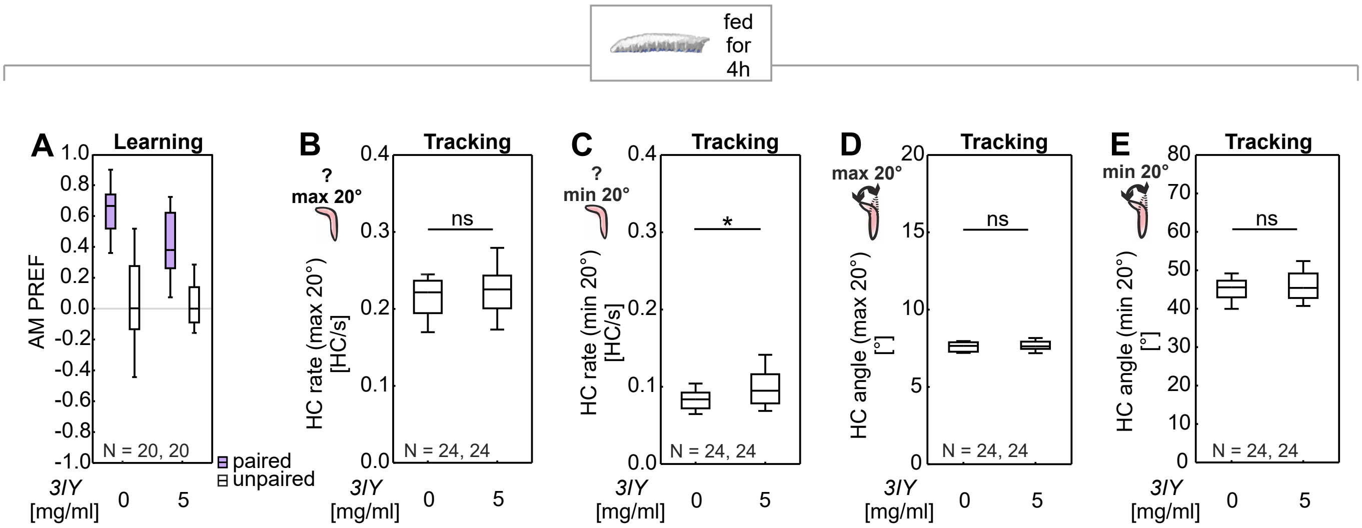

Fig. S2. Preference and tracking data underlying the results shown in Fig. 2. (A) Preference data refer to the Performance Indices shown in Fig. 2A. Purple boxes represent odor preference after paired training, white boxes after unpaired training. (B,C) HC rate classified by small and large HCs. The HC rate for small HCs was unaffected by 3IY feeding (B) (MWU: $U = 231.00$, $P = 0.2440$, N = 24 each), but slightly increased for large HCs (C) (MWU: $U = 186.00$, $P = 0.0364$, N = 24 each). Comparing these results with those after 24 h feeding (Fig. S1E-F), it seems that both 4 h and 24 h feeding of 3IY slightly increased the rate of large HCs, but only 24 h feeding decreased the rate of small HCs in addition. This resulted in a decrease in total HC rate after 24 h feeding on 3IY as seen in Fig. 1F, and in an increase in total HC rate after 4 h feeding on 3IY as shown in Fig. 2E. (D,E) HC angles classified by small and large HCs. No significant effect of 3IY feeding was observed (MWU: HC angle max 20°: $U = 256.00$, $P = 0.5160$, N = 24 each; HC angle min 20°: $U = 268.00$, $P = 0.6876$, N = 24 each). Asterisks and ns above horizontal lines reflect significance or lack thereof in MWU-tests. Box plots represent the median as the midline, 25 and 75% as the box boundaries, and 10 and 90% as the whiskers. Sample sizes are indicated within the figure.

Biology Open • Supplementary information

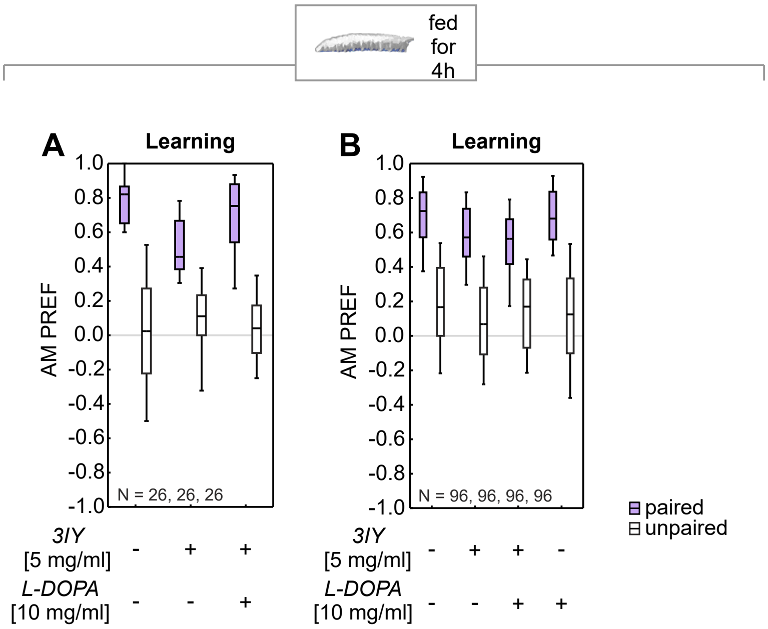

Fig. S3. Preferences underlying the Performance Indices in Figure 3. (A,B) Preference data refer to the Performance Indices shown in Fig. 3A,D, respectively. Purple boxes represent odor preference after paired training, white boxes after unpaired training. Box plots represent the median as the midline, 25 and 75% as the box boundaries, and 10 and 90% as the whiskers. Sample sizes are indicated within the figure.

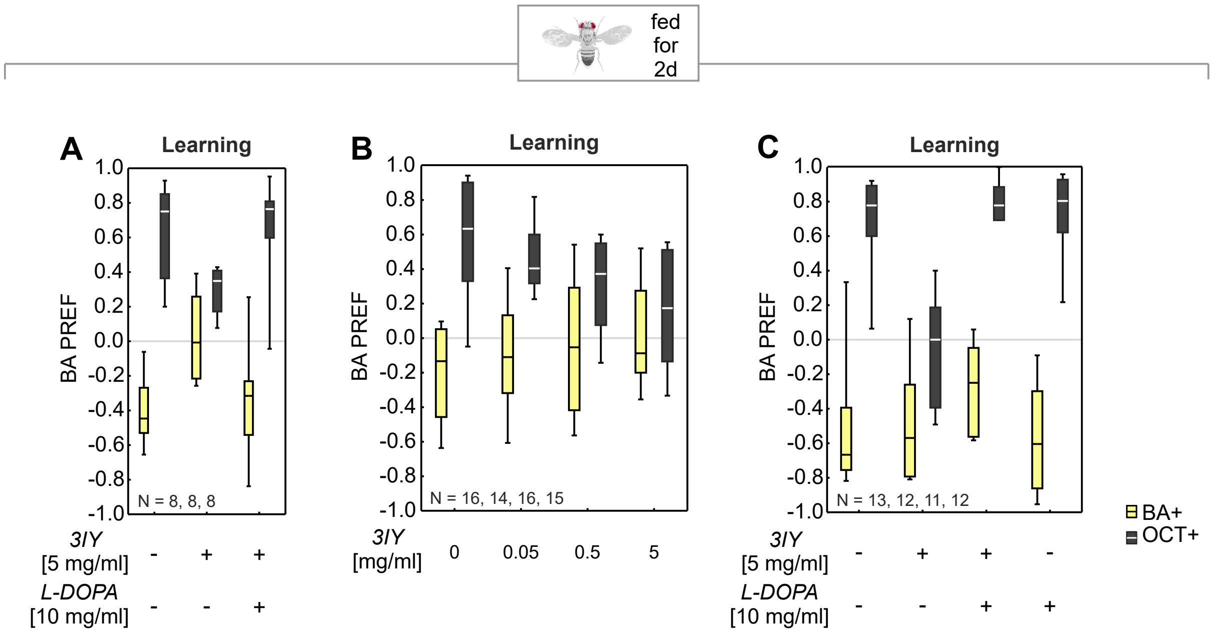

Fig. S4. Preferences underlying the Performance Indices in Fig. 4. (A,B,C) Preference data refer to the Performance Indices shown in Fig. 4B,C,G, respectively. Yellow boxes represent BA preference after BA-paired training (BA+), black boxes after OCT-paired training (OCT+). Box plots represent the median as the midline, 25 and 75% as the box boundaries, and 10 and 90% as the whiskers. Sample sizes are indicated within the figure.

Biology Open • Supplementary information

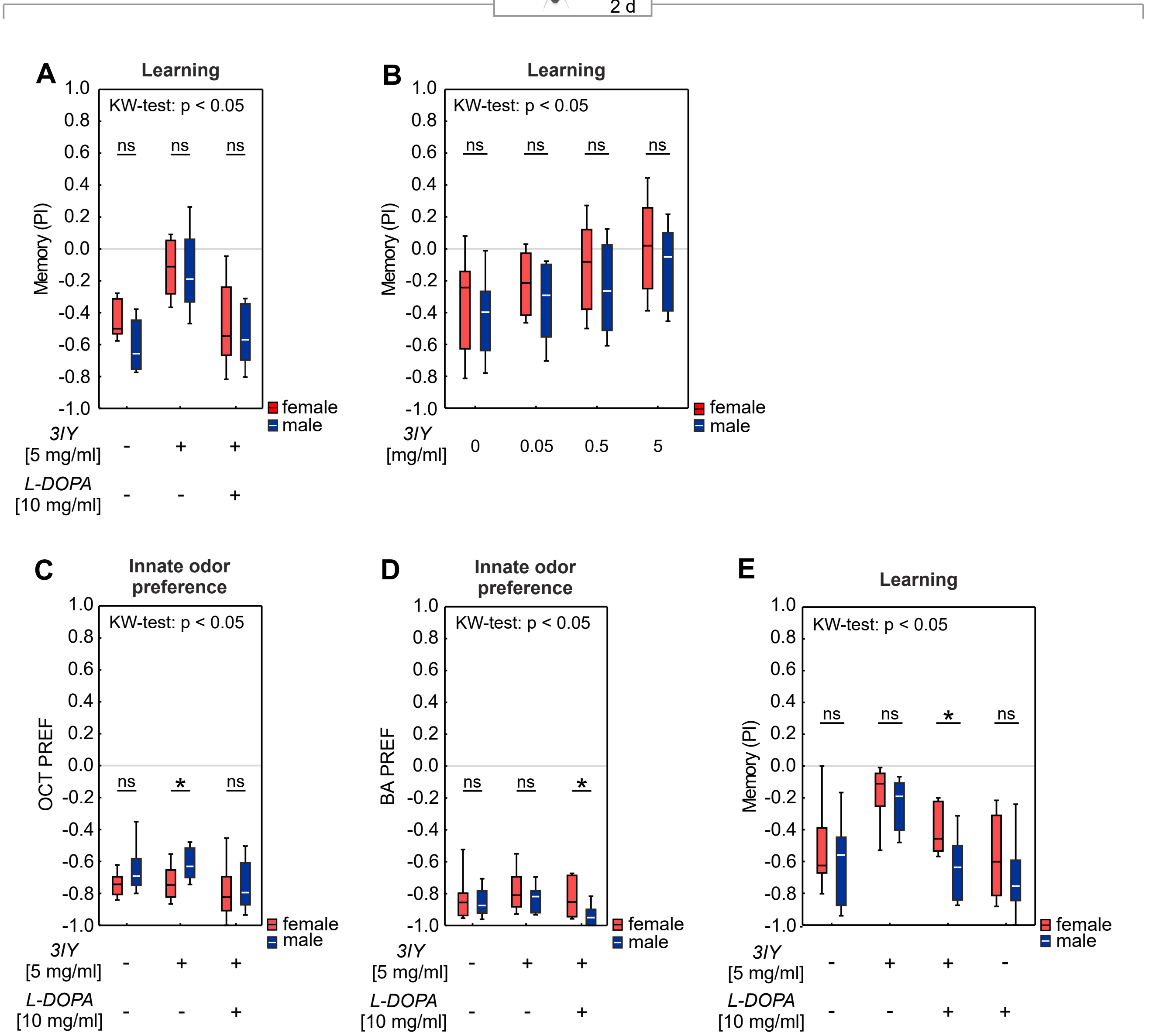
A
Learning
KW-test: p < 0.05
ns ns ns
Memory (PI)
female
male
3IY [5 mg/ml] - + +
L-DOPA [10 mg/ml] - - +
B
Learning
KW-test: p < 0.05
ns ns ns ns
Memory (PI)
female
male
3IY [mg/ml] 0 0.05 0.5 5
C
Innate odor preference
KW-test: p < 0.05
ns * ns
OCT PREF
female
male
3IY [5 mg/ml] - + +
L-DOPA [10 mg/ml] - - +
D
Innate odor preference
KW-test: p < 0.05
ns ns *
BA PREF
female
male
3IY [5 mg/ml] - + +
L-DOPA [10 mg/ml] - - +
E
Learning
KW-test: p < 0.05
ns ns * ns
Memory (PI)
female
male
3IY [5 mg/ml] - + + -
L-DOPA [10 mg/ml] - - + +

Fig. S5. Analysis of gender effects of data shown in Fig. 4. (A) Feeding of 3IY and/or L-DOPA had no gender specific effect (KW: H = 23.40, P = 0.0003; MWU: no drug: U = 16.00, P = 0.1036; 3IY alone: U = 31.00, P = 0.9581; 3IY + L-DOPA: U = 27.00, P = 0.6365; OSSs from left to right: P = 0.0078; P = 0.0078; P = 0.7266; P = 0.4531; P = 0.0078; P = 0.0078; N = 8 each). (B) No effects specific for gender with increasing concentration of 3IY feeding (KW: H = 20.53, P = 0.0045; MWU: control: U = 99.00, P = 0.2828; 0.05 mg/ml 3IY: U = 77.00, P = 0.3462; 0.5 mg/ml 3IY: U = 100.00, P = 0.2999; 5 mg/ml 3IY: U = 94.00, P = 0.4553; OSSs from left to right: P = 0.0042; P = 0.0005; P = 0.0225; P = 0.0018; P = 0.4545; P = 0.4545; P = 1.0000; P = 1.0000; N = 16, 16, 14, 14, 16, 16, 15, 15). (C) Only in the group fed with 3IY male flies showed slightly higher OCT preference than females (KW: H = 12.01, P = 0.0346; MWU: no drug: U = 48.50, P = 0.1842; 3IY alone: U = 36.00, P = 0.0404; 3IY + L-DOPA: U = 63.00, P = 0.6236; OSSs from left to right: P = 0.0005; P = 0.0010; P = 0.0005; P = 0.0005; P = 0.0005; P = 0.0005; N = 12 each). (D) Only in the group fed with 3IY and L-DOPA females showed slightly higher BA preference than males (KW: H = 11.81, P = 0.0375; MWU: no drug: U = 71.50, P = 1.0000; 3IY alone: U = 62.00, P = 0.5834; 3IY + L-DOPA: U = 29.00, P = 0.0141; OSSs: P = 0.0005 each; N = 12 each). (E) Only females fed with 3IY and L-DOPA show less punishment learning than males of the same group (KW: H = 30.58, P = 0.0001; MWU: no drug: U = 84.00, P = 1.0000; 3IY alone: U = 55.00, P = 0.9526; 3IY + L-DOPA: U = 23.00, P = 0.0151; L-DOPA alone: U = 56.50, P = 0.3865; OSSs from left to right: P = 0.0063; P = 0.0034; P = 0.0063; P = 0.0005; P = 0.0010; P = 0.0010; P = 0.0005; P = 0.0063; N = 13, 13, 12, 12, 11, 11, 12, 12). Red boxes represent females, blue boxes males. Box plots represent the median as the midline, 25 and 75% as the box boundaries, and 10 and 90% as the whiskers. Sample sizes are indicated within the figure.

Biology Open • Supplementary information

Table S1

Click here to download Table S1

Biology Open • Supplementary information
